# Subclassification of Newly Diagnosed Glioblastomas through an Immunohistochemical Approach

**DOI:** 10.1371/journal.pone.0115687

**Published:** 2014-12-29

**Authors:** Siobhan Conroy, Frank A. E. Kruyt, Justin V. Joseph, Veerakumar Balasubramaniyan, Krishna P. Bhat, Michiel Wagemakers, Roelien H. Enting, Annemiek M. E. Walenkamp, Wilfred F. A. den Dunnen

**Affiliations:** 1 Department of Pathology and Medical Biology (Division of Pathology), University of Groningen, University Medical Center Groningen, Groningen, The Netherlands; 2 Department of Medical Oncology, University of Groningen, University Medical Center Groningen, Groningen, The Netherlands; 3 Department of Neuroscience, University of Groningen, University Medical Center Groningen, Groningen, The Netherlands; 4 Department of Pathology, University of Texas, MD Anderson Cancer Center, Houston, Texas, United States of America; 5 Department of Neurosurgery, University of Groningen, University Medical Center Groningen, Groningen, The Netherlands; 6 Department of Neurology, University of Groningen, University Medical Center Groningen, Groningen, The Netherlands; University Hospital of Navarra, Spain

## Abstract

Molecular signatures in Glioblastoma (GBM) have been described that correlate with clinical outcome and response to therapy. The Proneural (PN) and Mesenchymal (MES) signatures have been identified most consistently, but others including Classical (CLAS) have also been reported. The molecular signatures have been detected by array techniques at RNA and DNA level, but these methods are costly and cannot take into account individual contributions of different cells within a tumor. Therefore, the aim of this study was to investigate whether subclasses of newly diagnosed GBMs could be assessed and assigned by application of standard pathology laboratory procedures. 123 newly diagnosed GBMs were analyzed for the tumor cell expression of 23 pre-identified proteins and *EGFR* amplification, together allowing for the subclassification of 65% of the tumors. Immunohistochemistry (IHC)-based profiling was found to be analogous to transcription-based profiling using a 9-gene transcriptional signature for PN and MES subclasses. Based on these data a novel, minimal IHC-based scheme for subclass assignment for GBMs is proposed. Positive staining for IDH1^R132H^ can be used for PN subclass assignment, high EGFR expression for the CLAS subtype and a combined high expression of PTEN, VIM and/or YKL40 for the MES subclass. The application of the proposed scheme was evaluated in an independent tumor set, which resulted in similar subclass assignment rates as those observed in the training set. The IHC-based subclassification scheme proposed in this study therefore could provide very useful in future studies for stratification of individual patient samples.

## Introduction

Glioblastoma (GBM) is the most frequent and aggressive primary brain tumor in adults with a 5-year survival rate of less than 10% [1]–[3]. Standard treatment comprises resection of the majority of tumor mass, followed by radiochemotherapy [3], [4]. The uniformly poor prognosis for all GBM patients merits improvements in treatment. To better understand GBM biology, several groups have therefore turned to high-dimensional profiling studies over the last decade. These studies were performed in large GBM patient cohorts and have identified a variety of GBM subclasses; the number of subclasses and their more precise defining features are, however, still under debate [5]–[8]. The Proneural (PN) and Mesenchymal (MES) subclasses have been identified most consistently and these signatures were applied to approximately 30% and 30 to 50% of GBMs, respectively [5], [6]. The PN subclass is characterized by mutations in isocitrate dehydrogenase 1 (*IDH1*), and frequent alterations in expression of tumor protein p53 (p53) and platelet-derived growth factor receptor, alpha polypeptide (PDGFR-a) [6], whereas the MES subclass is characterized by mesenchymal gene expression, of which CD44 and chitinase 3-like 1 (YKL40) are strong indicators [5], [6], [9], [10]. Other subclasses such as Classical (CLAS), Neural (N) and Proliferative (P) have also been proposed. The CLAS subgroup is characterized by alterations in the epidermal growth factor receptor (*EGFR*), and the N subclass by expression of neuronal markers. The P subclass shows high expression of proliferative markers like proliferating cell nuclear antigen (PCNA) and topoisomerase II alpha (TOP2A) [5], [6]. Many of the differential expression patterns are reflective of alterations in chromosomes 7, 9 and 10, and these alterations have been shown to have prognostic value in itself as well [11]–[14]. In this study we focus however only on the more easily accessible protein expression levels.

The clinical correlation of these molecular signatures has been subject of investigation in two cross-sectional studies on archival GBM material [5], [6]. One of these studies reported significantly better survival by the PN group, and they described the MES group as the poorest prognosis group [5], whereas another study only identified a trend towards better survival for the PN group [6]. The latter study also described better response to more intensive therapy by the CLAS and MES group, whereas this was not observed for the PN group. To identify novel types of therapy targeting alterations specific for molecular subclasses it will be important to implement molecular subclassification in future clinical trials.

An advantage of subclassification at protein level is that it does not require additional types of tissue processing than those already performed for normal diagnostic procedures. In addition, high-throughput techniques are costly and less feasible for individual patients in daily practice, thereby hindering inclusion of molecular subtyping in laboratory and clinical studies. Therefore, this study was set up to evaluate the possibility of subtyping by immunohistochemistry (IHC) and fluorescence *in situ* hybridization (FISH) on formalin-fixed paraffin-embedded (FFPE) GBM tissue. To assess this, a selection of 123 newly diagnosed GBM cases was analyzed for the expression of 23 pre-identified proteins and *EGFR* amplification on a tissue microarray (TMA). Next, unsupervised hierarchical clustering was performed to identify groups of tumors with similar expression patterns. Then, to relate the newly identified IHC/FISH-based molecular profiles to previously described transcriptional profiles, tumors representative of the PN or MES IHC/FISH-based profiles were assessed for a 9-gene PN/MES-transcriptional signature [15]. Ultimately this study proposes a novel scheme for IHC-based subclass assignment, which is evaluated in an independent GBM tumor set. This novel scheme can be applied for the individual patient sample using only a small selection of antibodies.

## Materials and Methods

### Patient population

Tissue samples were collected of adult patients that had received a diagnosis of GBM (World Health Organization Grade IV astrocytoma) from November 2005 to July 2012 at our institution (department of Pathology, University Medical Center Groningen, Netherlands). The patient selection was limited to newly diagnosed GBMs. Tumors with radiological evidence of a less malignant precursor lesion, so-called secondary or progressive GBMs, were excluded from the study. Patients who underwent neurosurgical debulking and of which sufficient paraffin-embedded archival tumor material was left after diagnosis were eligible for the study. Finally, tumor tissue of 123 patients was included. The mean patient age at diagnosis in the study group was 60 years (range 23–80; Table 1) and a two-third overlap (70% of patients) was noted with the expected peak incidence age group of primary GBM (45 to 70 years) [16].

**Table 1:** Summary of characteristics of patients with newly diagnosed GBM in the training and validation tumor set.

Characteristic	Training tumor set	Validation tumor set
Number of patients	123	44
Mean age at diagnosis (range)	60 (23–80)	62 (32–84)
Male sex (%)	73 (59)	26 (59)
Female sex (%)	50 (41)	18 (41)

Additionally, an independent validation tumor set consisting of 44 patients was assembled following the same eligibility criteria as were applied for the training set, but these patients were diagnosed with GBM from August 2012 to January 2014 at our institution. The mean patient age in this tumor group was 62 (range 32–84, Table 1), which also had a substantial overlap (77% of patients) with the peak incidence age group of primary GBM.

All experiments including the use of human tissue were conducted under the ‘Code of Conduct for dealing responsibly with human tissue in the context of health research' published by the Federation of Dutch Medical Scientific Societies in 2011 [17]. The human tissue used in this study was originally obtained for diagnostic purposes and the scientific research performed on the leftover material is considered to be ‘further use’ of the tissue. The authors had no interaction with the participants and all samples were anonymized before the authors obtained them. Research on coded-anonymous human tissues is deemed acceptable under an augmented system of opt-out when no specific circumstances apply, and therefore approval was not required for this study.

### Tissue microarray (TMA)

Sections from the original FFPE tissue blocks of the surgical debulkings were stained with H&E and reviewed by a neuropathologist (WdD) to allow for selection of vital tumor areas, after which biopsies were punctured from these sites. From all tumors 4 biopsies of a 0.6 mm diameter were incorporated in the TMAs and positive control tissue was also added directly in the TMAs. This control tissue concerned samples from cortex, white matter, cerebellum, colon, endometrial, kidney, liver, and spleen. The control tissue was used as a technical reference in the evaluation of the staining patterns of the markers applied in this study. Paraffin sections of 3 µm thickness were prepared for immunostaining and 5 µm sections for FISH.

### Selection of markers specific of GBM subclasses

The selection of potential markers of interest for IHC/FISH-based subclassification of GBM, was started with the markers from Phillips' and Verhaak's classification of GBMs [5], [6]. This collection of markers was narrowed down by selecting those markers for which experience with GBM IHC-analysis was previously reported. Additionally, markers assessed in separate papers for which relevance for subclassification of GBMs was suggested were also included. Together this resulted in a final selection of 23 GBM subclass-associated markers suitable for IHC analysis and a single FISH marker.

### Immunofluorescence (IF) staining

Delta-like 3 (DLL3) expression was assessed by IF staining [9]. Endogenous peroxidase of deparaffinized GBM tissue on TMAs was blocked using routine techniques. The tissues were blocked for 30 minutes with 3% normal rabbit serum in 1% BSA/PBS and then incubated with anti-DLL3 (diluted 1∶200 in 1% BSA/PBS; S1 Table) for 1 hour at room temperature (RT). The tissues were then incubated for 30 minutes at RT with peroxidase-conjugated rabbit anti-goat serum (DAKO, Glostrup, Denmark) diluted 1∶100 in 1% BSA/PBS. The final incubation step was one hour at RT with donkey anti-rabbit fluorescently labeled antibody (Alexa fluor 647, Invitrogen, Karlsruhe, Germany) diluted 1∶200 in 1% BSA/PBS. Nuclear counterstaining by DAPI (diluted 1∶1000) was added to the mounting medium (Fluorescence Mouting Medium, DAKO).

### IHC

Deparaffinized GBM tissue on TMAs was used to evaluate the expression of several proteins. Antigen retrieval methods specified per antibody are listed in S1 Table. Endogenous peroxidase was blocked using 0.3% H_2_O_2_ for 30 minutes at RT. The tissues were then incubated with a primary antibody at RT for 1 hour, followed by application of a secondary antibody (peroxidase-conjugated serum, DAKO), and a tertiary antibody (peroxidase-conjugated serum, DAKO), each for 30 minutes at RT. Antibody details of the primary antibodies used in this study are listed in S1 Table. The secondary and tertiary antibodies were diluted 1∶100 in 1% BSA/PBS with 1% AB serum. Color development was performed with 3,3′-diaminobenzidine for 10 minutes, and tissues were counterstained with haematoxylin for 2 minutes.

### Histological evaluation

Exclusively tumor cells were evaluated for the expression of proteins. The scoring method was tailored to the observed staining pattern for individual markers, thereby leading to six different semi-quantitative scoring schemes for the IHC evaluation. Representative micrographs of the scoring patterns applied to each individual staining are depicted in S1 and S2 Figs.

Markers reflecting genetic alterations (EGFRvIII, and *IDH1* R132H mutation [IDH1^R132H^]) were scored as either 0 =  “no alteration”, or 1 =  “altered”. Also the EGFR pattern was evaluated best by scoring as either 0, no expression, or 1, corresponding to diffuse high expression. A single score was assigned to each tumor for *IDH1* ^R132H^ and EGFR based on all 4 cores as the expression of these markers was consistent in each of the cores within a tumor. For all other markers described the final score given for a marker was the result of the average of the four biological replicates.

For two markers (fox-1 homolog 3 [NeuN] and oligodendrocyte lineage transcription factor 2 [OLIG2]) a previously suggested scoring system was applied [9]. Briefly, scoring was performed in 4 categories per core to quantify the number of tumor cells expressing the respective antigen (0: no expression; 1: <10% expression; 2: 10%–49% expression; 3: 50%–90%).

Markers that displayed complete negative cores, cores with mixed positive and negative sites, and complete positive cores were scored as 0, 1 and 2, respectively (chromogranin A [CHGA], fibronectin 1 [FN1], neurofilament light chain [NEFL], p53, phosphatase and tensin homolog [PTEN], and vimentin [VIM]).

For three markers (CD44, hepatocyte growth factor receptor [MET], and synaptophysin [SYP]) an additional intermediate level was required, thus resulting in a scoring scheme range from 0 to 3. The scores for these markers were 0 for a complete negative core, 1 for focal weak staining, 2 for diffuse weak staining or focal intense staining, and 3 was assigned to diffuse intense staining.

Besides these expression patterns, there were also markers that did not show complete negative expression (glial fibrillary acidic protein [GFAP], nestin [NES], neurofibromin 1 [NF1], PDGFR-a, signal transducer and activator of transcription 3 [STAT3], and YKL40). These markers were scored either 1, basal level expression, or 2, diffuse intense expression of the marker.

Finally, the proliferation index of individual tumors was assessed by two antibodies (Ki-67 and TOP2A). Overview pictures of histological slides were scanned using a C9600 NanoZoomer (Hamamatsu Photonics KK, Hamamatsu City, Japan) and analyzed using ImageScope software (Aperio, Vista, CA). Squares were placed within the individual cores to delineate an area containing 100 tumor cells at minimum. In this block the total number and the positively stained tumor cells were counted, allowing for calculation of the positive fraction (proliferation index).

### FISH

Dual-probe FISH analysis was performed on 5 µm sections of the TMAs. Standard laboratory procedures were applied, and for hybridization the Vysis *EGFR*/*CEP7* FISH Probes were used (Abbott Molecular, Des Plaines, IL). Briefly, deparaffinized slides were prepared for probe hybridization, including a boiling step, incubation with RNA'se A solution and 0.1% pepsin solution. After probe hybridization the tissues were dehydrated, and nuclei were counterstained using DAPI diluted 1∶3000 in mounting medium (Vectashield, Vector Laboratories, Burlingame, CA).

As there are currently no solid criteria for scoring of *EGFR* FISH in GBM available, but suggestions have been postulated in a recent paper [18]. We adopted these criteria, and shortly summarized, tumors were assigned a positive FISH status when the ratio of *EGFR* to centromere (*CEP7*) signal was ≥2, and a negative FISH status was assigned in events where the *EGFR∶CEP7* ratio was <2. Representative images are shown in S2 Fig.

### Cluster analysis

Data were analyzed using Genesis v1.7.6 [19]. Prior to clustering the scoring range of all markers was standardized, except for the markers that directly or indirectly reflected genetic abnormalities (*EGFR* FISH, EGFRvIII, and IDH1^R132H^). Hierarchical clustering was performed, using Kendall's tau coefficient and average-linkage as clustering method, and clustering was applied to both markers and tumors.

### PN/MES index

A PN/MES index was calculated based on protein expression scores in a comparable manner as was described previously [9]. The selected PN markers were IDH1^R132H^ and p53, and the MES markers were PTEN, VIM, and YKL40. For each individual marker scoring was performed as described under histological evaluation. The scores for IDH1^R132H^ and p53 were recoded into scores ranging from 0 to 1.5, and the MES scores were recoded into scores ranging from 0 to 1. The PN and MES subtype specific markers were then added up to obtain a total PN and MES score, both having a maximum total score of 3. Subsequently the PN/MES index was calculated by subtraction of the MES total score from the PN total score.

### Quantitative Reverse Transcriptase-Polymerase Chain Reaction (qRT-PCR)

RNA was purified from 20 FFPE GBM samples as described previously [20]. To assess purity of tumor tissue in these archival samples, sections of 3 µm thickness were prepared of all GBM samples, and stained with H&E. Purified RNA was quantified using Nanodrop ND-1000 UV spectrophotometer (NanoDrop Technologies, Wilmington, DE). The RNA samples were DNase treated using the Ambion turbo DNA Free kit (Life Technologies, Bleiswijk, the Netherlands) to remove contaminating genomic DNA, and thereafter RNA content was again quantified using Nanodrop. RNA was reverse transcribed to single-stranded cDNA using Superscript II reverse transcriptase (Invitrogen) and random hexamer primers (Promega, Leiden, the Netherlands). The qRT-PCR was performed in triplicate with the qPCR MasterMix Plus (Eurogentec, Maastricht, the Netherlands), using an ABI7900HT real-time sequence detection system in 384-well reaction plates. The following assays, all TaqMan single-gene expression assays (Applied Biosystems, Foster City, CA), were used: ASCL1 (Hs00269932_m1), CHI3L1 (Hs00609691_m1), DLL3 (Hs01085096_m1), NCAM1 (Hs00941821_m1), OLIG2 (Hs00377820_m1), RPS27 (Hs01378332_g1), SERPINE1 (Hs01126606_m1), TGFB1 (Hs00932747_m1), and TIMP1 (Hs00355335_g1). The qRT-PCR data were analyzed using SDS software version 2.2.2.

In order to determine the association of the IHC/FISH-based molecular profiles identified in this study with previously described transcriptional profiles [5], [6], 20 patient samples characteristic of either the PN (n = 10) or the MES (n = 10) subclass in the IHC/FISH-based analyses were evaluated by a qRT-PCR fingerprint. In this analysis, RPS27 was used as reference gene; ASCL1, DLL3, NCAM1, and OLIG2 were used as PN genes and CHI3L1, SERPINE1, TGFB1, and TIMP1 as MES genes. The averaged C_T_-value of the reference gene was subtracted from C_t_-values of the genes of interest, resulting in normalized expression values (ΔC_t_-values) for individual PN and MES genes.

Secondly, a metagene score was calculated to generate a comparative heatmap [15]. By averaging the inverse of the ΔC_t_-values for the PN and MES genes, application of Z-score correction, and finally subtraction of the PN score from the MES score, a metagene score was generated.

### Statistical analysis

Differences between groups were determined by the Mann-Whitney U or Kruskal-Wallis test using SPSS software version 20.0.02 (SPSS, Chicago). Significant Kruskal-Wallis tests were followed up by post-hoc tests (Bonferroni corrected). Correlations were calculated by Pearson's r or Spearman's rho. *P*-values <0.05 were considered significant.

## Results

### Subclass-specific marker expression in newly diagnosed GBMs

The PN subclass was examined through antibodies specific for DLL3, IDH1^R132H^, OLIG2, p53, and PDGFR-a. All PN markers, except DLL3, showed sufficient differences in their staining pattern to allow semi-quantitative scoring (S1 and S2 Figs.). For that reason DLL3 was excluded from further protein expression analyses in the present paper. The mutation specific antibody for *IDH1* ^R132H^ [21]–[23], detecting a mutation frequently found in lower-grade gliomas [23]–[25], generated an easily distinctive pattern in our sample of GBMs. A total of 6 of 123 tumors (5%) were found to be positive for the R132H mutation in *IDH1*. Detectable p53-expression by IHC analysis was observed in 119 of 123 tumors (97%), but diffuse high overexpression of p53 (indicative of mutation) was observed in only 19 of 123 tumors (15%). Elevated PDGFR-a expression was observed in 27 of 123 tumors (22%). Overall 43 of 123 tumors (35%) showed one or more positive PN markers.

EGFR markers were included to represent the CLAS subtype [5], [6]. Expression of EGFR and its constitutively active (mutant) form EGFRvIII were assessed by IHC, while *EGFR* amplification was assessed through FISH. The expression level of EGFR and EGFRvIII was for all tumors consistent in all cores. Out of 123, 51 tumors (41%) presented with elevated EGFR protein expression. *EGFR* amplification was observed in 49 tumors (40%) and EGFRvIII expression was observed in 24 of 123 tumors (20%). Substantial concordance of the EGFR-positive tumors was with the *EGFR* amplification status (Spearman's rho [SR]  = 0.765, *P*<0.01) and EGFRvIII expression (SR = 0.543, *P*<0.01), and also between the *EGFR* amplification status and EGFRvIII expression (SR = 0.521, *P*<0.01). The IHC analysis for EGFR identified 43 of the 49 tumors in which *EGFR* amplification was observed. Among these double positive tumors for EGFR protein expression and gene amplification were 23 of the total 24 tumors in which EGFRvIII expression was identified.

The MES subclass was assessed through expression of the markers CD44, FN1, NES, NF1, STAT3, VIM, and YKL40 [5], [7], [9], [10], [26]–[31]. High expression of 2 or more MES markers was found in 51 of 123 tumors (41%). Immunological markers, even though previously associated with the MES subclass, were omitted from the current study [9]. Such markers can be used to assess activation or infiltration of the immune system, but the tumor cells themselves mostly do not express these markers. That renders TMA-based analysis unsuitable for these markers.

The expression of neuronal markers in GBM and their potential role in subclassification has been described [32]–[34]. The expression of neuronal markers CHGA, NeuN, NEFL, and SYP was therefore also assessed in this study. High expression of at least one of the neural markers was observed in 38 of 123 tumors (31%), whereas 14 of these 38 tumors (37%) had coinciding expression of at least two neuronal markers. Interestingly, mature neuronal cells, possibly healthy cells trapped in the tumor, were also found to be present in several tumors.

For the Proliferative subclass Ki-67 and TOP2A were included as representative markers [5]. Proliferative indices calculated from these stainings varied from 1.3% to 88.2% for Ki-67, and from 0% to 97.4% for TOP2A. The correlation between these proliferation markers was significant (Pearson r = 0.542, *P*<0.001).

Finally, three markers with reported variable association with different subclasses were also included in the study. This concerned MET, previously associated with the PN or the MES subclass [6], [35], GFAP, variably associated with the PN, MES or Neural subclass [5], [33], [36], and PTEN, formerly mentioned to associate with the PN or the MES subclass [5], [6], [35]. In our sample 18 tumors (15%) displayed high MET expression, 26 of 123 tumors (21%) had high GFAP expression, whereas PTEN was highly expressed by 29 of 123 tumors (24%).

### Identification of three distinct subclasses through unsupervised hierarchical clustering

**Figure 1:**
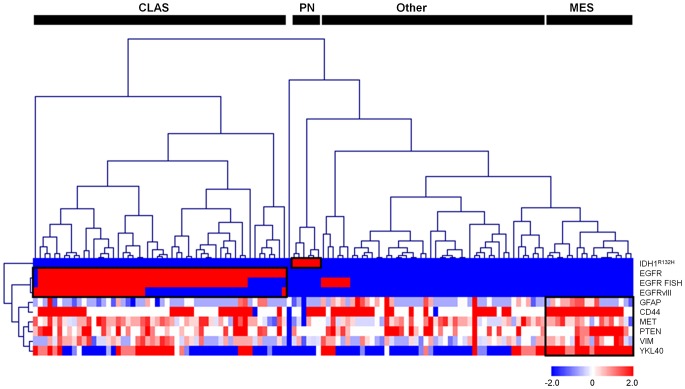
Hierarchical clustering of 123 newly diagnosed GBMs reveals three distinct expression patterns. The expression patterns of 9 proteins and the FISH status of *EGFR*, all positively or negatively associated with one of the predefined GBM subclasses (PN, CLAS, or MES), are depicted in the heat map. Kendall's tau coefficient is displayed as a similarity measure. This figure provides evidence for a differential IHC/FISH-based expression pattern by the three subclasses.

The markers with differential and reproducible staining patterns were used to cluster the complete set of 123 GBM samples. Unsupervised hierarchical clustering of both markers and samples was performed, which resulted in the identification of three distinct IHC/FISH-based clusters (Fig. 1). In the clustering process, markers without discriminating power were excluded from the study at two instances. The first instance for marker exclusion was when the markers did not result in a differential staining pattern in the tumor set prior to clustering (DLL3 only). Second, markers that did not show any cluster association in the first clustering attempt were removed from the study because they did not cluster with their pre-designated cluster (FN1, NES, p53 and PDGFR-a, S3 Fig.). Markers were also excluded in this step because they did not identify a specific group with high expression of these markers (CHGA, Ki-67, NeuN, NEFL, OLIG2, SYP and TOP2A). In the second clustering attempt NF1 and STAT3 showed very low association with the MES markers and were excluded.

The first delineated cluster of tumors expressed markers characteristic of the CLAS subtype (52 tumors, 42%; Fig. 1). Elevated EGFR protein expression was observed in 51 of 52 tumors (98%), while *EGFR* amplification was diagnosed in 43 of 52 tumors (83%). Expression of EGFRvIII, indicative of the deletion of exon 2 to exon 7 from the *EGFR* gene, was observed in 24 of 52 tumors (46%). All EGFR markers were expressed significantly more in the CLAS group than in other subclasses (*P*<0.001). Interestingly, a small group of 6 tumors exhibited EGFR gene amplification but without protein overexpression.

The second cluster covered 6 tumors (5%) all expressing mutant IDH1 (R132H, PN; Fig. 1). The *IDH1* ^R132H^ mutation was not found in the other clusters (*P*<0.001). Besides this *IDH1* mutation, high p53 expression was also detected more frequently in the PN tumors. EGFR alterations and mesenchymal protein expression were mutually exclusive with this cluster, while high expression of neural markers was observed for half of the PN tumors. This cluster showed high similarities to the PN subclass identified in previous studies [5], [6].

**Figure 2:**
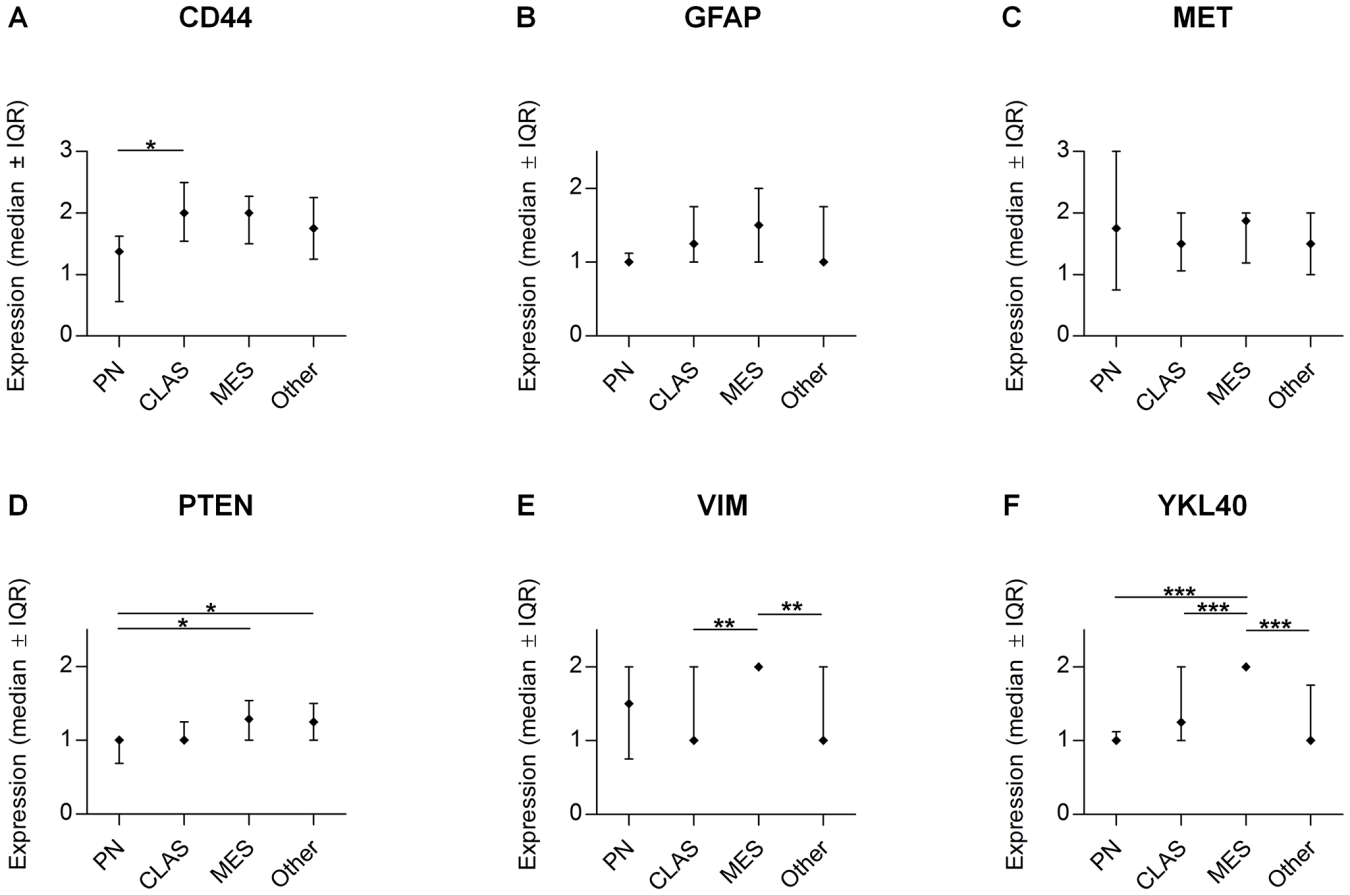
Marker validation graphs of the discriminating markers indicative of the MES subclass. Mean protein expression of CD44 (**A**), GFAP (**B**), MET (**C**), PTEN (**D**), VIM (**E**) and PTEN (**F**) in the molecular subclasses is displayed. Values represent mean ± SEM; * *P*<0.05; ** *P*<0.01; *** *P*<0.001.

The third cluster (18 tumors, 15%), concerned tumors with high expression of a combination of MES markers (MES; Fig. 1). A cluster was formed by PTEN, VIM, CD44, GFAP, MET, and YKL40, most of them with differential expression in the molecular subclasses (Fig. 1). In Fig. 2 the quantification of the expression levels of MES markers is illustrated and compared over the identified subclasses. It can be appreciated in this figure that only the MES markers PTEN, VIM and YKL40 are able to discriminate specifically the MES group from the other subclasses based on their median expression level. These markers are therefore considered the most discriminative markers.

Taken together, the IHC/FISH-based subclassification system was able to assign predefined subclasses to 76 of 123 tumors included in the present study. The PN and CLAS subclasses were characterized best by markers related to gene mutations, gene amplifications or changes in protein expression, while the MES was classified best by simultaneous high protein expression of several markers. The previously described P and N subtypes were not detected as distinct subclasses in this analysis.

### PN/MES index

**Figure 3:**
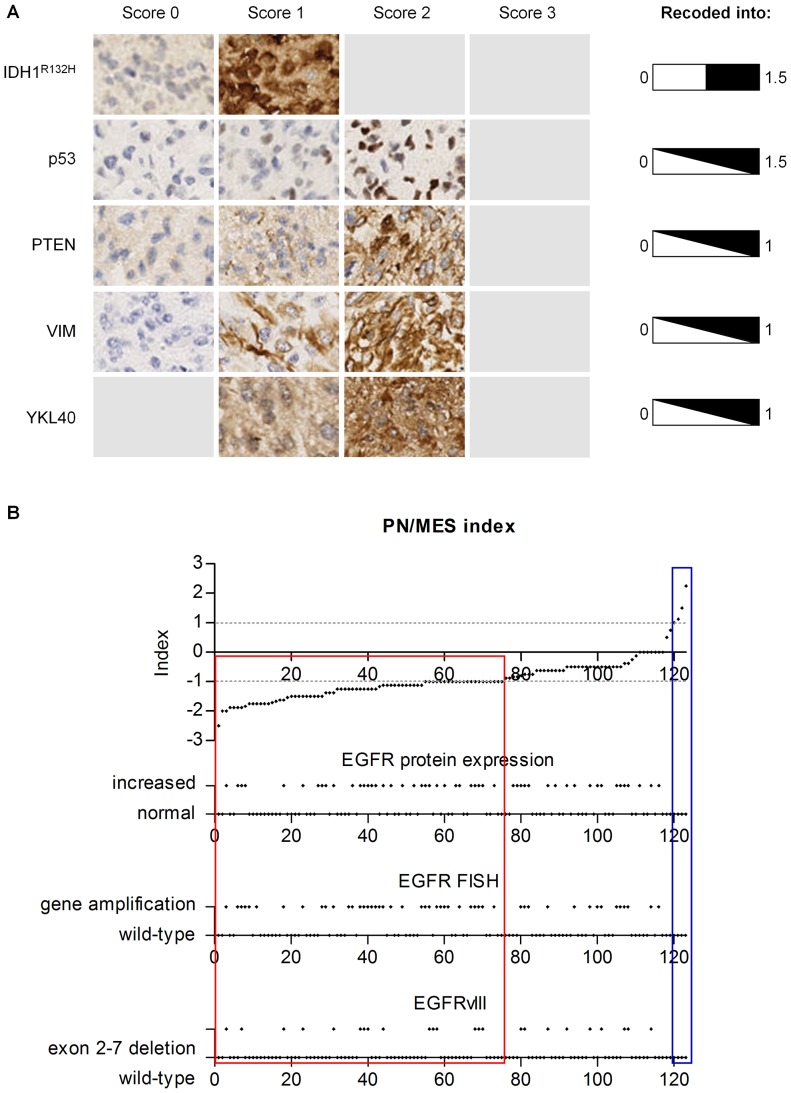
IHC-based GBM subclassification into PN and MES subclasses based on two (PN) or three (MES) representative stainings for each subclass. **A.** Representative photomicrographs (100x magnification) for PN and MES GBM markers (PN: IDH1^R132H^ and p53; MES: PTEN, VIM, and YKL40) are shown. The recoding method of the stainings is also indicated. **B.** The distribution of the tumors based on the PN/MES index is depicted here. Tumors with a PN/MES index greater than 1 were assigned the PN subclass (blue box), while a PN/MES index smaller than −1 were assigned the MES subclass (red box). The distribution of CLAS markers (EGFR expression level, *EGFR* amplification, and an EGFR mutation [EGFRvIII]) was aligned with the index scores.

The feasibility of IHC/FISH-based PN and MES subclassification was also assessed by the calculation of a so-called PN/MES index. The most discriminating markers for the PN (*IDH1* ^R132H^ and p53) and MES (PTEN, VIM and YKL40) subclass based on our results were used to calculate this index, in line with a previously described method (Fig. 3A) [9]. A clearly distinctive PN group was identified on the right side of the graph (PN/MES index >1, n = 3), and a MES group on the left side of the graph (PN/MES index <−1, n = 75). The other 44 tumors were not assigned any specific subclass (Fig. 3B).

As this index was merely able to assign the PN and MES subclass to tumors and other GBM subclasses are known to exist, a considerable amount of tumors was on forehand expected to not be assigned any subclass. To assess in which region of the graph these other subclasses were actually present, we next plotted characteristic features of the CLAS subtype below the PN/MES index. Tumors positive for CLAS features were distributed almost randomly along the graph with a slight increase among the lower PN/MES indices, but they were completely absent among the tumors characterized as PN in this analysis (Fig. 3B). Chi-Square analyses pointed out that the distribution of CLAS features was statistically not different among the classified groups in this analysis. This was the case for EGFR expression (*X^2^* [2, n = 123]  = 2.93, *P*>0.05), *EGFR* amplification status (*X^2^* [2, n = 123]  = 5.29, *P*>0.05) and EGFRvIII expression (*X^2^* [2, n = 123]  = 1.01, *P*>0.05).

### IHC/FISH-based GBM subclasses are analogous to transcriptional subclasses

**Figure 4:**
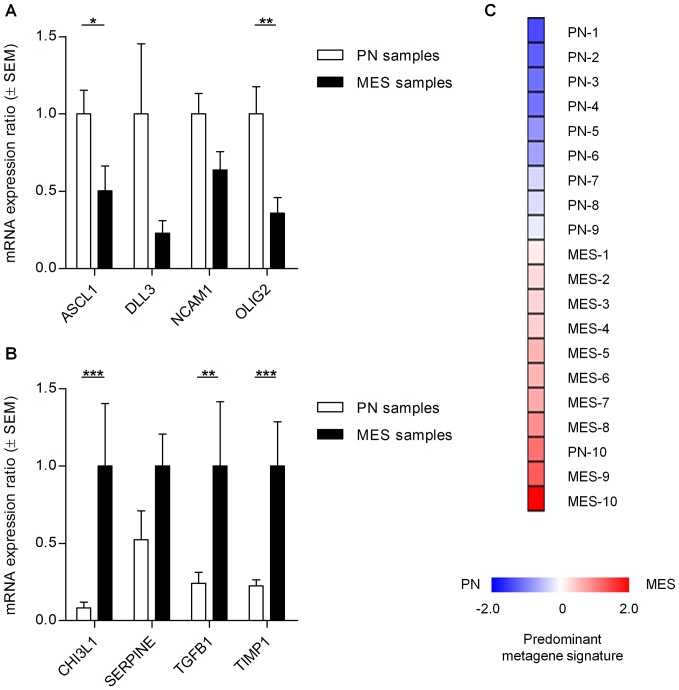
Transctiption-based PN/MES gene expression profiles are in line with PN and MES IHC/FISH-profiles. **A.** Expression ratios of mRNA of four PN genes (*ASCL1, DLL3, NCAM1, and OLIG2*) by core PN and MES samples (n = 10 per subclass) are depicted. Significant higher expression of PN genes by core PN samples was observed for two PN genes. The expression level by PN samples was set at 1. **B.** Expression ratios of mRNA of four MES genes (*CHI3L1*, *SERPINE*, *TGFB1*, and *TIMP1*) by core PN and MES samples (n = 10 per subclass) are depicted. Significant higher expression of MES genes by core MES genes was observed for three of four MES genes. The expression level by MES samples was set at 1. (**C**) Heatmap of Z-score corrected metagene scores, calculated on the basis of the expression of the PN and MES genes. Blue shades represent a predominantly PN signature, red a MES signature, whereas white shades represent a relatively balanced expression of both signatures as indicated in the figure. Values represent mean ± SEM; * *P*<0.05; ** *P*<0.01; *** *P*<0.001.

To substantiate the relation of IHC/FISH-based PN and MES subclasses to previously established transcriptional profiles [5], we performed qRT-PCR on core samples from the PN and MES subclasses identified by the cluster analysis and the PN/MES index. Four well-established PN and four MES genes [15] were assessed to compare the gene expression of these genes in the IHC/FISH-based PN and MES group (Fig. 4A and B, S2 Table). The mRNA expression ratios were compared and 2 out of 4 PN genes were found to be expressed significantly higher in the IHC/FISH-characterized PN group, while 3 of 4 MES genes were found to be expressed significantly higher in the IHC/FISH-characterized MES group.

Secondly, a metagene score was calculated by a previously described method [15]. An almost complete separation of the IHC/FISH-based PN samples from the IHC/FISH-based MES samples was observed (Fig. 4C), but PN sample 5 was found to transcriptionally have a more MES than PN profile. Taken together, this implies that GBM samples assigned the PN subclass in the IHC/FISH study corresponded to the established transcriptional PN subclass, and samples assigned the MES subclass corresponded to the established transcriptional MES subclass.

### Validation of IHC-based subclassification of newly diagnosed GBMs

To further validate the potential of IHC-based subclassification of newly diagnosed GBMs, an additional set of newly diagnosed GBMs was analyzed for the expression of the markers that proved most useful in the analyses described in this study. In this tumor set 2 tumors (5%) were positive for *IDH1* ^R132H^. A total of 21 of 44 tumors (48%) presented with elevated EGFR expression, and only 1 of the EGFR-positive tumors was positive for EGFRvIII expression. The expression of MES markers PTEN, VIM and YKL40 was also assessed, allowing for the detection of high expression of minimally 2 MES markers in 16 of 44 tumors (55%).

**Figure 5:**
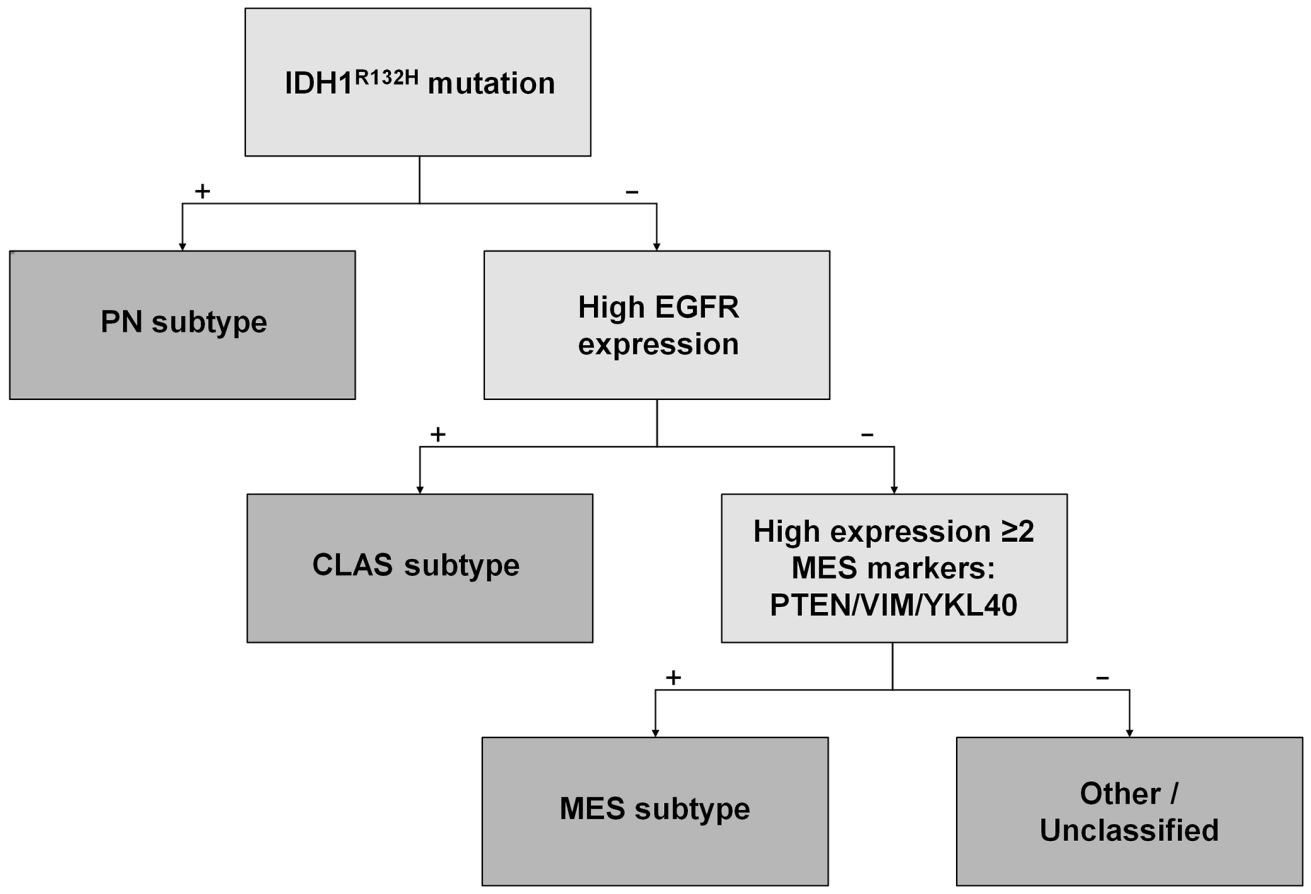
Proposed scheme of IHC-based subclass assignment in newly diagnosed GBMs. In the first step the PN subtype is assigned on the basis of the R132H mutation in *IDH1* . In the second step the CLAS subtype is assigned on the basis of high EGFR expression. In the final step the MES subtype is assigned in case two or more MES markers (PTEN, VIM and YKL40) are coincidently expressed at a high level.

By first assigning the PN subclass, then assessing the expression of CLAS features to assign this subclass, and finally assigning the MES subclass based on expression of MES markers, IHC-based subclasses were assigned to the validation tumor set. Application of this scheme resulted in classification of 2 tumors (5%) as PN, 21 tumors (48%) as CLAS and 7 tumors (16%) as MES. The assignment rates for the subclasses are summarized in Table 2, where it can be observed that the subclass assignment rates are highly similar in the training and validation tumor set. A summary of the proposed assignment scheme is displayed in Fig. 5.

**Table 2:** Subclass assignment rates (%) per subclass in the training and validation tumor set.

Subclass	Training tumor set	Validation tumor set
	n = 123	n = 44
PN	5	5
CLAS	42	48
MES	15	16
Other/Unclassified	38	31

## Discussion

In the current study, the feasibility of GBM subclassification by the use of tools available in routine diagnostic settings was assessed. Three of the predefined subclasses could be identified through an unsupervised hierarchical cluster analysis of 10 pre-selected subclass-specific markers. Based on our results we propose a minimal selection of markers required for IHC/FISH-based subtype assignment. For the PN subclass *IDH1* ^R132H^ was found to be the only discriminative marker, as the previously suggested markers PDGFR-a and p53 did not show association with the PN subclass. A separate cluster characterized by positivity for these two markers, which would resemble the *IDH1-*wild-type PN tumors described more recently [37], were not identified either. The CLAS subtype was characterized best by the IHC analysis for EGFR, and most of the CLAS tumors were also detected by the EGFRvIII IHC analysis. For MES subclass assignment expression analyses for PTEN, VIM and YKL40 were found to be superior to other MES markers. The application of multiple markers for the MES subclass is necessary as the differential expression of these markers even within the MES subclass can be appreciated in Fig. 1. Subclass-assignment by the novel model we propose in this study (Fig. 5) was evaluated in an independent tumor set leading to similar assignment rates as those observed in the training tumor set.

Besides selection of subclass-discriminative markers, the order of subclass assignment is also important in the newly proposed model. As the defining features of the PN signature are mutually exclusive with both the markers for the CLAS and MES signature, the first subclass to be assigned should be the PN. In the second step the CLAS subclass should be assigned, because the CLAS markers do not appear in the pure MES subclass. In the final step the MES tumors can be distinguished from tumors that cannot be assigned a specific subclass. We emphasize the importance of the order of subclass assignment and the application of multiple markers where appropriate, as tumors classified as CLAS here could have easily been assigned the MES subclass in a different order of subclass assignment. Coinciding expression of markers from several subclasses is known not to affect *IDH1*-mutant PN tumors [15], but MES marker expression in CLAS tumors does occur often [38]. The order of assignment proposed here will result in more reliable and reproducible subclasses than would be the case when employing previously suggested classification schemes [39]–[41]. These studies do not describe an assignment scheme, do not include all the subclasses or do not assess the order of subclass assignment.

The profiling through the 9-gene transcriptional PN/MES signature revealed that IHC/FISH-based PN and MES signatures is comparable to the previously established transcriptional signatures. All the differences in expression ratios of subtype-matched genes were in the expected direction, although the actual differences between these signatures are expected to be larger than reported here. As only 6 tumors were characterized as PN in the cluster analysis (Fig. 1), the PN group was complemented with tumors assigned the highest PN/MES indices (Fig. 3B). One of these additional tumors transcriptionally showed a high MES score (PN-10 in Fig. 4C). When looking at the protein expression profile of this sample we also find that p53 expression was high, but high expression of multiple MES markers like PTEN, VIM was also documented for this sample. The fact that YKL40 was not highly expressed in this sample explains the more PN-like score from the IHC-based PN/MES index. Application of the scheme proposed in Fig. 5 would have led to classification of this tumor as MES, underscoring the better functionality of our proposed scheme in comparison to the previously described IHC-based scheme for PN/MES subclassification [9].

The ratio of subclass assignment to tumors in this study (62%) was considerably lower than in previous studies. In one study the complete patient group was subclassified [5], and in the other study 85% of the tumor set was subclassified (due to exclusion of 29 of 202 cases for reasons of lower similarity to the identified subclasses) [6].

At this point it cannot be ruled out that the PN tumors in our study sample concern a small group of secondary GBMs, which did not cause symptoms in lower-grade stages and were only recognized when they had progressed into the GBM state. As secondary GBMs are histologically undistinguishable from primary GBMs, it is impossible to diagnose this retrospectively. The defining characteristics of the PN subtype are also frequently found in lower-grade gliomas or secondary GBMs [42]–[44], and mutations in *IDH1* and *TP53* are normally found in 70% and 50 to 60%, respectively, of lower-grade astrocytomas [43], [45]–[47]. Previous studies have not excluded lower-grade astrocytoma (grade III) from their patient sample (21 of 76 tumors) [5], or the patient sample also included secondary or recurrent GBMs (19 of 202 tumors) [6]. The rate of PN signature occurrence amongst newly diagnosed GBMs thus seems debatable, and in agreement with the suggestion by Nousmehr et al. [37], the exclusion of secondary GBMs in our study resulted in a low assignment rate of the PN subclass. The exclusion of secondary GBMs thus selected against the *IDH1*-mutant PN tumors, also referred to as the G-CIMP-positive tumors, but the lack of markers for the G-CIMP-negative PN tumors probably also contributed substantially to the lower identification rate of the PN subclass [37], [48], [49].

The rate of assignment of the CLAS subtype in our study (42%) was slightly higher than in other reports, whereas MES subclass assignment was in our study considerably lower than in others [5], [6]. The MES subclass, contrary to the PN subclass, is merely characterized by markers associated with *de novo* GBM. At least in the case of the MES subclass inclusion criteria can thus not serve as an argument for the ratio-difference of subclass assignment in our study. We propose that the co-expression of multiple molecular signatures within tumors could provide an explanation. Also in previous studies at the transcriptional level subsets of tumors expressed markers characteristic of different subtypes, but in all these cases the tumors possessed a dominant expression pattern for only one of the subclasses [5], [6]. Tumors lacking dominant expression of a single molecular signature, as was found for several tumors in our study, have not been described in transcriptional studies before, possibly explaining the overall lower rate of subclass assignment in this study. The finding of co-occurrence of multiple molecular signatures is however supported by a recent report, which described the co-existence of a PN-like subset of cells (*PDGFRA* amplification) besides a CLAS-like subset of cells (*EGFR* amplification) within a single tumor, which was determined through FISH-analysis [50]. Recognition of these mixed expression patterns thus seems compromised at transcriptional level, but can be appreciated by FISH and protein expression analyses.

Provided that approximately two thirds of tumors (76 of 123, 62%) were subclassified into the three predefined subclasses in our study, the question how useful subclassification for GBMs in fact is, could be posed. Clearly, a considerable number of patients will not benefit from this subclassification effort, as they will not be assigned any of the subtypes described in this report. We expect that the benefit of the subclassification, however, lies in the fact that a restricted number of patients (one of the subtypes) benefiting from a treatment could now be recognized in a clinical trial, while such survival advantages for a subset of patients would likely have been missed in a non-stratified study. Given the enormous heterogeneity of GBMs, identifying a subgroup of patients benefiting from a treatment is probably a more realistic and expectable advancement than a uniform cure for all GBM patients.

## Conclusion

In conclusion, we were able to subclassify two-thirds of GBMs into predefined molecular subclasses through the assessment of protein expression patterns. We found that multiple signatures within the same tumor could be detected at protein level, whereas this has not been described at the transcriptional level. The IHC-based signatures identified in this study did however strongly resemble the established transcriptional profiles. Subclassification of an independent tumor set resulted in similar subclass assignment rates as were reported for the training tumor set.

The identification of more effective therapies from which only a part of GBM patients benefits requires that tumor subclassification is incorporated in preclinical and clinical studies. Incorporation of subclassification will also enable development of therapies targeted at shared alterations within GBM subclasses. Our suggested method, making use of techniques widely available in standard diagnostic settings of pathology departments and by application of only a minimal selection of markers, could provide very useful for subtyping of GBM samples of individual patients in future studies.

## Supporting Information

S1 Fig **Representative micrographs and scoring schemes of PN and MES markers previously reported to have GBM subclass-associated expression patterns.** Micrographs were obtained at 100x magnification.
(TIF)

S2 Fig **Representative micrographs and scoring schemes of CLAS, N and other markers previously reported to have GBM subclass-associated expression patterns.** Micrographs were obtained at 100x magnification, FISH at 1000x magnification.
(TIF)

S3 Fig **Initial result of hierarchical clustering of 123 newly diagnosed GBMs using 23 protein markers and 1 FISH analysis.** Groups of tumors and clusters of markers can be appreciated and are highlighted with black boxes. The P and N markers are clustered in separate groups, but did not identify isolated groups of tumors with high expression of these markers as primary characteristic. Kendall's tau coefficient is displayed as a similarity measure; *: Markers did not cluster with their predesignated clusters.
(TIF)

S1 Table Antigen retrieval methods and primary antibodies used for IHC and IF analyses.
(DOCX)

S2 Table qRT-PCR data (n = 20) used to calculate the 9-gene PN/MES signature.
(DOCX)
