# Supplementary figures and images for: Subclassification of Newly Diagnosed Glioblastomas through an Immunohistochemical Approach
Source: PLoS One. 2014 Dec 29;9(12):e115687. doi: 10.1371/journal.pone.0115687 (PMC4278713; doi:10.1371/journal.pone.0115687)

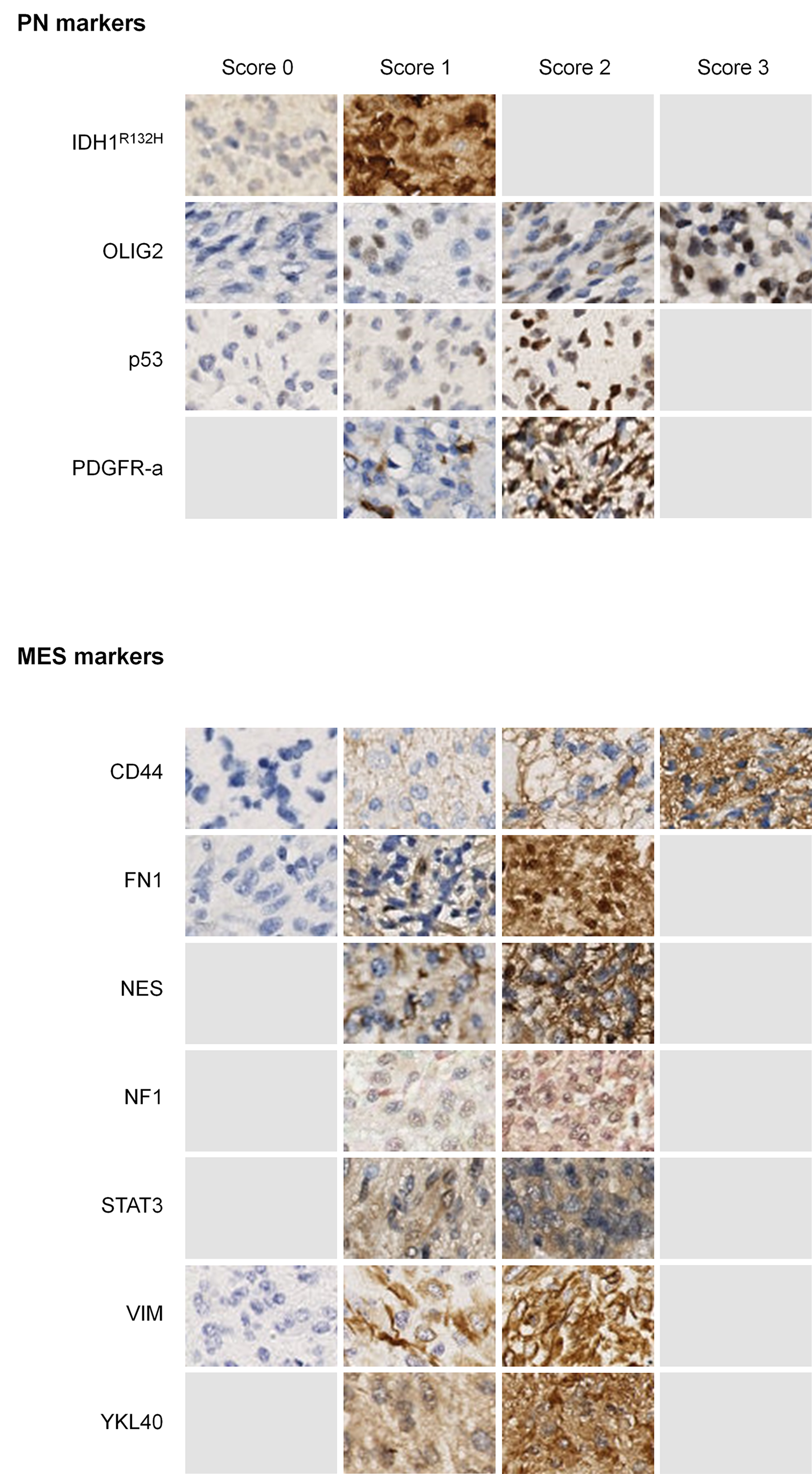

Supplement: S1 Fig — Representative micrographs and scoring schemes of PN and MES markers previously reported to have GBM subclass-associated expression patterns. Micrographs were obtained at 100x magnification. (TIF) [file pone.0115687.s001.tif]

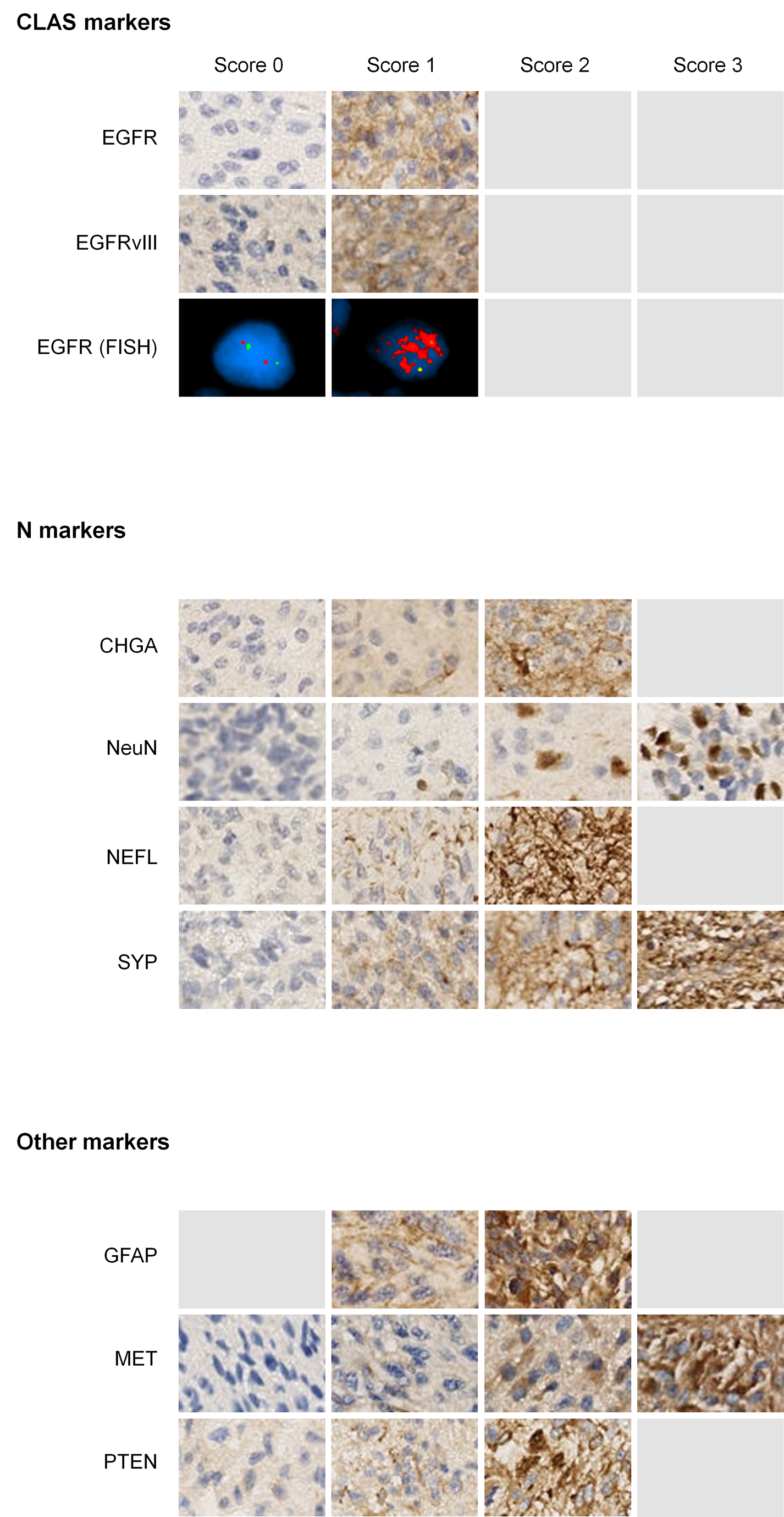

Supplement: S2 Fig — Representative micrographs and scoring schemes of CLAS, N and other markers previously reported to have GBM subclass-associated expression patterns. Micrographs were obtained at 100x magnification, FISH at 1000x magnification. (TIF) [file pone.0115687.s002.tif]

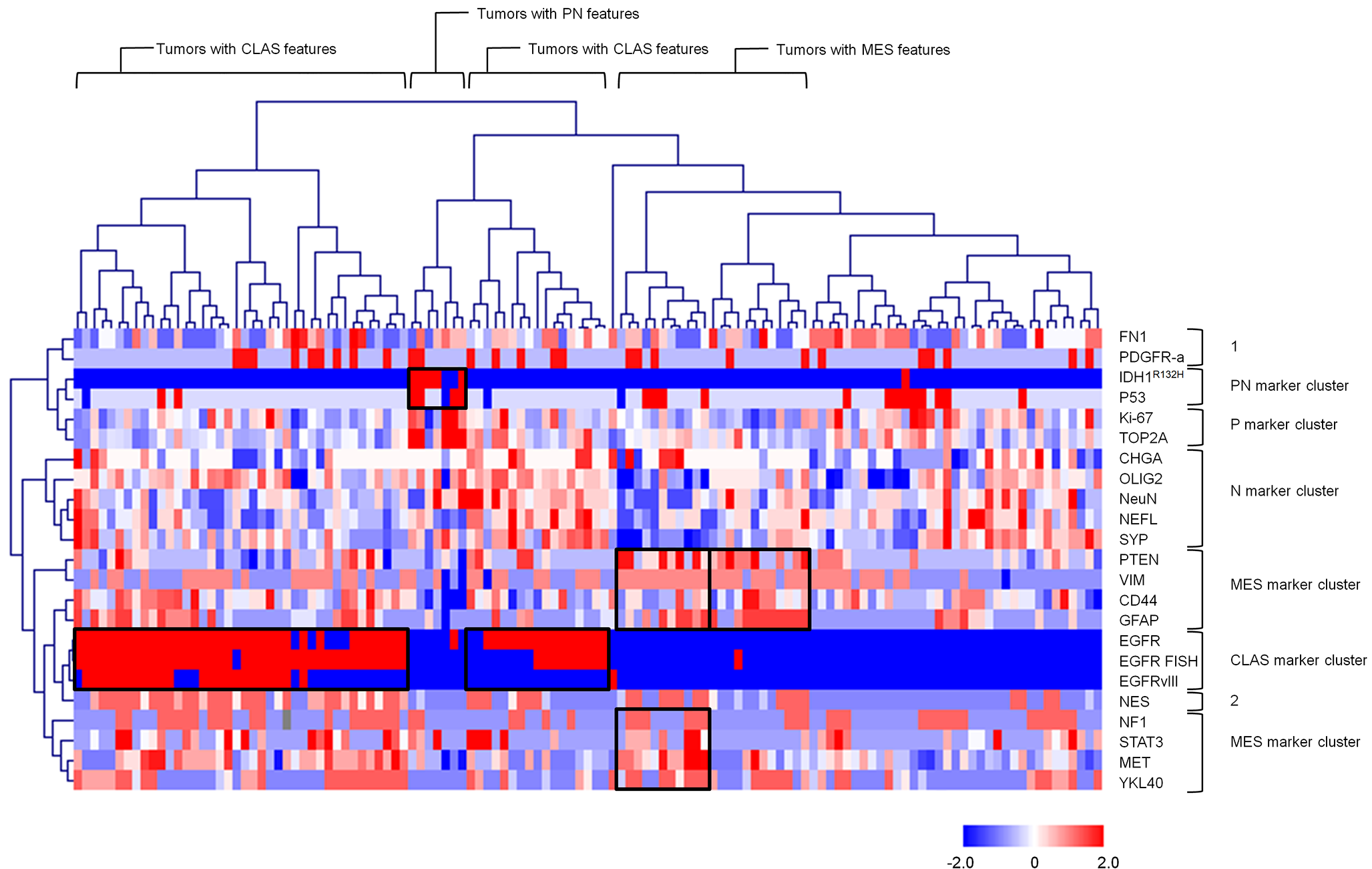

Supplement: S3 Fig — Initial result of hierarchical clustering of 123 newly diagnosed GBMs using 23 protein markers and 1 FISH analysis. Groups of tumors and clusters of markers can be appreciated and are highlighted with black boxes. The P and N markers are clustered in separate groups, but did not identify isolated groups of tumors with high expression of these markers as primary characteristic. Kendall's tau coefficient is displayed as a similarity measure; *: Markers did not cluster with their predesignated clusters. (TIF) [file pone.0115687.s003.tif]
