# Supplementary material for: Subclassification of Newly Diagnosed Glioblastomas through an Immunohistochemical Approach
Source: PLoS One. 2014 Dec 29;9(12):e115687. doi: 10.1371/journal.pone.0115687 (PMC4278713; doi:10.1371/journal.pone.0115687)
Supplement: S1 Table — Antigen retrieval methods and primary antibodies used for IHC and IF analyses. (DOCX) [file pone.0115687.s004.docx]

**Table S1.** Antigen retrieval methods and primary antibodies used for IHC and IF analyses.

| **Antibody** | **Origin** | **Target** | **Dilution** | **Antigen retrieval** | **Source** |
| --- | --- | --- | --- | --- | --- |
| CD44 | Mouse (mAB) | 80-kDa Transmembrane glycoprotein | 1:200 | C | 3c11; Cell Signaling, Danvers, MA |
| CHGA | Rabbit (pAB) | Chromogranin A; parathyroid secretory protein 1 | 1:1000 | T/E | A0430; DAKO, Glostrup, Denmark |
| MET | Rabbit (mAB) | Receptor for hepatocyte growth factor | 1:200 | C | 51067; Abcam, Cambridge, UK |
| DLL3 | Goat (pAB) | Delta-like 3 (drosophila) | 1:100 | - | C-17; Santa Cruz, Heidelberg, Germany |
| EGFR | Mouse (mAB) | Epidermal Growth Factor Receptor | 1:100 | T/HCl | EGFR113; Monosan, Uden, The Netherlands |
| EGFRvIII | Mouse (mAB) | Epidermal Growth Factor Receptor variant III (constitutively active) | 1:200 | E | L8A4; kindly provided by Dr Bigner, Duke University, Durham, NC |
| FN1 | Rabbit (pAB) | Fibronectin type 1 | 1:100 | C | 2413; Abcam |
| GFAP | Rabbit (pAB) | Glial Fibrillary Acidic Protein | 1:250 | - | Z0334; DAKO |
| IDH1^R132H^ | Mouse (mAB) | Isocitrate dehydrogenase 1 R132H mutation | 1:40 | T/E | H09; Dianova, Hamburg, Germany |
| Ki-67 | Mouse (mAB) | Marker of proliferation Ki-67 | 1:300 | T/E | MIB-1; DAKO |
| NES | Mouse (mAB) | Nestin | 1:100 | C | 10c2; Santa Cruz |
| NeuN | Mouse (mAB) | RNA binding protein, fox-1 homolog 3 | 1:100 | T/E | A60; Merck Millipore, Billerica, MA |
| NEFL | Mouse (mAB) | Neurofilament light chain (68 kDa) | 1:20 | T/E | 2F11; DAKO |
| NF1 | Rabbit (pAB) | Neurofibromin | 1:100 | C | 30325, Abcam |
| OLIG2 | Rabbit (pAB) | Oligodendrocyte lineage transcription factor 2 | 1:100 | C | 18953; Immuno-Biological Laboratories, Gunma, Japan |
| p53 | Mouse (mAB) | Tumor protein p53 | 1:1000 | T/E | DO-7; DAKO |
| PDGFR-a | Rabbit (pAB) | Platelet derived growth factor receptor, alpha polypeptide | 1:200 | C | C-20; Santa Cruz |
| PTEN | Mouse (mAB) | Phosphatase and tensin homolog | 1:100 | C | 6H2.1; Cascade bioscience, Winchester, MA |
| STAT3 | Rabbit (pAB) | Signal transducer and activator of transcription 3 | 1:200 | C | 9132; Cell Signaling |
| SYP | Mouse (mAB) | Synaptophysin | 1:50 | T/E | 27G12; Monosan |
| TOP2A | Mouse (mAB) | Topoisomerase II alpha | 1:750 | C | Ki-S1; DAKO |
| VIM | Mouse (mAB) | Vimentin | 1:100 | P | 3B4; DAKO |
| YKL40 | Goat (pAB) | Chitinase 3-like 1 | 1:200 | C | S18; Santa Cruz |

*mAB* monoclonal antibody, *pAB* polyclonal antibody, *C* citrate buffer, *E* EDTA buffer, *T/E* Tris/EDTA buffer, *T/HCl* Tris/HCl buffer, *T/HCl 80ºC* Tris/HCl buffer overnight 80ºC, *P* 0.1% protease
