# Supplementary material for: Subclassification of Newly Diagnosed Glioblastomas through an Immunohistochemical Approach
Source: PLoS One. 2014 Dec 29;9(12):e115687. doi: 10.1371/journal.pone.0115687 (PMC4278713; doi:10.1371/journal.pone.0115687)
Supplement: S2 Table — qRT-PCR data (n = 20) used to calculate the 9-gene PN/MES signature. (DOCX) [file pone.0115687.s005.docx]

**Table S2.** qRT-PCR data (n=20) used to calculate the 9-gene PN/MES signature.

| **Genes** | **Average ΔC_t_ values (±SEM)** | | |
| --- | --- | --- | --- |
|  | **PN patients (n=10)** | **MES patients (n=10)** | **All patients (n=20)** |
| ASCL1 | 5.67 (±0.27) | 7.05 (±0.51) | 6.28 (±0.31) |
| DLL3 | 5.75 (±0.65) | 7.29 (±0.57) | 6.43 (±0.47) |
| NCAM1 | 4.11 (±0.23) | 4.87 (±0.31) | 4.49 (±0.21) |
| OLIG2 | 3.48 (±0.33) | 5.46 (±0.62) | 4.47 (±0.42) |
| CHI3L1 | 5.20 (±0.97) | 0.41 (±0.48) | 2.80 (±0.76) |
| SERPINE | 3.82 (±0.40) | 2.73 (±0.40) | 3.28 (±0.30) |
| TGFB1 | 2.41 (±0.48) | 0.23 (±0.40) | 1.32 (±0.39) |
| TIMP1 | 3.71 (±0.25) | 1.71 (±0.29) | 2.71 (±0.30) |
